# The Adjustment Disorder Diagnosis, Its Importance to Liaison Psychiatry, and its Psychobiology

**DOI:** 10.3390/ijerph16234645

**Published:** 2019-11-22

**Authors:** James J. Strain

**Affiliations:** Icahn School of Medicine at Mount Sinai, 1425 Madison Avenue, New York, NY 10029, USA; jim_strain@hotmail.com

**Keywords:** adjustment disorder, sub-threshold diagnoses, minor depression, biological underpinnings, measurement issues

## Abstract

Adjustment Disorder (AD) is one of the most common psychiatric diagnoses employed. In fact, it is the most frequent diagnosis utilized for psychiatric disorders in the military and in children, and is often utilized in the consultation-liaison medical setting. However, it is acknowledged that the diagnosis is not reliable, it cannot be validated, and it has an important degree of subjective consideration in its use. Commonly used screening tools like the Hamilton and Beck Depression Scales do not give an assessment of AD. Furthermore, its use is accompanied with descriptors of depression, anxiety, mixed affects, etc., so that it crosses over several areas of psychiatric dysfunction. It does allow the placement of a patient within a psychiatric diagnosis when they do not reach criteria for a major psychiatric nomenclature. To date, biological studies have not been reported. It is not known if AD with depression is closer to the biological characteristics of depression, or AD with anxiety would have similar characteristics to that seen with major anxiety. It is also not known if AD has a biological signature that would make them an entity with common features, or if they may be more closely allied biologically with the descriptor that accompanies them. Nevertheless, AD is an important category in any psychiatric lexicon and warrants further study and biological understanding.

## 1. Introduction

Liaison psychiatry are the newest specialty in American Psychiatry. In fact, there is an Academy of Consultation-Liaison Psychiatry that has enlisted hundreds of members to practice, research, and teach in this sub-specialty. The hallmark of liaison psychiatry is its involvement with the medically, surgically, ill, obstetrics-gynecology, and all the other medical specialties. There is a special group of psychiatrists that work with pediatric patients who usually have had a liaison fellowship in the pediatric setting. The focus of liaison psychiatry is the psychological care of the medically ill [1].

The need for a new sub-specialty arose from the psychological problems that accompany medical illness and their caretakers. The comorbidity of psychiatric and medical dysfunction impacts on the practitioner who must work in multiple medical specialties, deal with the medical-surgical setting and its demands, and needing knowledge and competence with medical drugs and their interaction with psychiatric medications. This new brand of psychiatry often involves undergoing a fellowship for one or two years located in a medical setting where all the patients have a medical-surgical disorder. There is also a separate set of literature and several journals for this new sub-specialty. Liaison psychiatry encompasses consultation psychiatry, but moves well beyond it.

Another additional set of knowledge, skills, and attitudes are essential for the liaison component of this work in the medical setting, where the goal is the education and enhancement of the psychological knowledge of the medical and nursing staff to identify, diagnose, and treat or refer a psychiatric disability in their patients. The psychiatrist becomes part of the team, is on the ward or in the medical setting, e.g., in the ambulatory clinic or primary care physician’s office on a regular basis, makes rounds on patients and works with the doctors, the nurses, and the social worker to enhance their capacity to identify and manage the psychological issues that medically ill patients present. This important teaching function is an attempt to not take over the patient, but help the medical caretaker to diagnose and manage their patient’s psychological issues themselves, as part of their practice. That would mean once out of the teaching hospital, or after sufficient collaborative care experience and training, the medical doctor would be able to diagnose and treat non-complicated psychological issues in his/her patient on their own, and know when to refer if they have a patient with a refractory or complicated illness, e.g., depressive disorder unresponsive to usual and customary doses of antidepressant medications, encounter someone with an unclear diagnosis, a patient needing complex psychotherapy and/or chemotherapy, or an actively suicidal patient.

For example, primary care physicians currently prescribe 70% of the antidepressant medication and 80% of the anti-anxiety medications in the US. It has been recently articulated that the primary care physician should be able to care for the “garden variety” depression and see it as a systemic illness as much as a psychological dysfunction [2,3,4]. In addition, it is essential to be cognizant of the systemic-biological-happenings with depression, e.g., the effects on the hypothalamic-pituitary-adrenal cortical activity (HPA), adrenals, glucose, immune system, and platelets, that in turn might affect the medical illness of their patient. Finally, the primary care physician should be able to refer those depressed patients who are refractory to attempts with one to three families of our most commonly employed antidepressant medications after adequate time for them to be effective at adequate dosages. It is important to know when and how to refer to a psychiatrist or mental health worker for those who remain refractory to “*garden variety*” interventions. Since most primary care physicians will not have a psychiatrist in their office, and they are prescribing these medications anyway, they should be conversant with diagnostic skills, treatment capacity, and referral techniques to those mental health professionals who are able to treat those refractory to their administrations. The most critical step is that the physician identify that their patient has a mental disorder and attempt to document its presence.

Liaison psychiatry also promotes the use of the team and ancillary personnel to deliver key information and treatment to the patient: the nurse, the social worker, the psychologist. In addition, the family or primary care takers need to be informed of the patient’s psychological problem with guidance on its management once the patient is at home. This also entails helping the primary care physician revise how he/she organizes their office to incorporate not only the use of the electronic health record (EHR) and screening devices, but also ancillary personnel, e.g., office secretary and nurse, to develop the psycho-social data base, and employing a social worker or psychologist to provide mental health care, e.g., cognitive behavioral therapy (CBT), psychotherapy, or family therapy, since that is not a skill the doctor would necessarily have, nor would he have time to deliver the talking cures [3,4].

Furthermore, the primary care physician may not be able to bill for this extra use of his/her time for psychological diagnoses and treatment. Strain describes in detail the use of medical informatics techniques to help identify and treat depression in the medical setting [4]. This includes the establishment of protocols for obtaining significant data and screening for depressive disorders, but not necessarily for the AD which unfortunately does not have a reliable or valid screening devise. An important change from Diagnostic and Statistical Manual -IV (DSM-IV) to Diagnostic and Statistical Manual -5 (DSM-5) was to eliminate the diagnosis of minor depression, which was a step closer to the AD in requiring only two major symptoms versus five for the full diagnosis of a major depressive disorder (MDD) [5,6]. Unfortunately, we do not have a valid and reliable screening device for AD [5,6].

## 2. Liaison Psychiatry and Adjustment Disorders (AD)

AD is one of the most commonly employed diagnoses in the medical-surgical setting. It is the most common diagnosis in the military and very frequently diagnosed in children. There are concerns about the validity of the diagnosis in so much as its three key diagnostic elements are subjective in nature and do not have metrics established for their presence or severity: (1) significant stressor, (2) feeling distressed and or, (3) evidencing dysfunction. According to the Diagnostic Statistical Manual -5 (DSM-5) of the American Psychiatric Association, a stressor is required that causes distress and/or dysfunction [6]. Neither the degree or nature of the stressor, the amount of distress, or the amount of dysfunction has been quantified in the key taxonomy used in the United States or the DSM-5, which renders this most commonly used diagnosis neither valid nor reliable. The DSM-5 does state that the reaction to the stressor is not in keeping with the amount and nature of the stressor or in line with what is expected in the culture of the particular individual, but there is no scale or metrics to assist in making the diagnosis.

In contrast, the proposed diagnostic requirements required for an AD diagnosis according to the International Classification of Disease -11 (ICD-11) includes all three entities: the stressor, dysfunction and distress, not either dysfunction or distress [7,8]. The importance of this is that in research studies different cohorts of patients will be different depending upon the criteria employed. Yet, again there are no specific measurements or metrics for any of the three entities central to an AD diagnosis in the proposed ICD-11 criteria. This means the two major psychiatric diagnostic taxonomies of the world do not agree on what constitutes an AD diagnosis, and neither have measurable guidelines for the assessment of its symptoms.

The ICD-11 group has further proposed that AD should include preoccupations with the stressor or its consequences, e.g., involuntary stressful reminders, or excessive worrying/ruminating about the event, and failure to adapt symptoms, e.g., avoidance or repression of feelings and thoughts about the stressful event (Maercker 2016) [9]. There is no universal measure employed for the screening of AD as there is for a MDD such as the Hamilton, or the Beck [10,11]. However, there is a disorder-specific questionnaire that has been developed by Einsle et al., that has not been universally accepted to assess AD [9]. The other concern is that it has not been employed in diverse patient cohorts.

This divergence in criteria of the primary lexicons to reach a diagnosis of AD is an important component of the difficulty that is confronted in research on this most frequently diagnosed disorder. The lack of standardized criteria and agreed upon metrics for a diagnosis of this disorder undermines the selection of research candidates for clinical trials. Moreover, studies of AD within the medically ill populations add an additional complication because of the different symptoms medical illnesses may present, e.g., the cognitive changes that may accompany HIV, the helplessness of a cerebral vascular accident, etc.

This remains the most important issue in finding homogeneous cohorts that are consistent across institutions for research purposes, and avoiding using diverse or heterogeneous groups of patients to attempt to understand the psychobiology and other properties of AD. There is at times great difficulty distinguishing normality from pathology, and no place in the psychiatric lexicon is this greater than in those disorders that have been regarded as sub-threshold. Furthermore, non-AD psychiatric and medical comorbidity need to be eliminated in the cohorts under study to ensure that there is a homogeneous diagnostic profile in the patients under investigation (Strain 2018) [12].

Therefore, it is difficult to conduct research studies of AD in the medical setting where diverse medical diagnoses and dysfunctions can differentially affect the presence and nature of an AD. None the less, it is a most useful diagnosis and is an identifier for a large group of patients who could and do benefit from mental health care. Because so many patients in the liaison setting do not reach the criteria for a major psychiatric disorder, but are experiencing distress and/or dysfunction, it is an appropriate diagnosis—with a reimbursable code—that can justify payment for important mental health interventions. Otherwise many persons with mental problems would not be identified and consequently would not be treated. Without a diagnostic code the physician could not bill for his/her efforts to care for these patients.

It is also important to note that AD is an important diagnosis for consultation-liaison psychiatry which deals with two genres of medicine: medical, surgical, ob-gyn—the medical patients; and psychiatry—the mental health field—the psychologically affected patients. The stressor in medical patients is most often the medical illness itself, which the patient has psychological reactions to, e.g., worries, fears, concerns, dependency, helplessness, pain, etc. The dysphoria is most often the mental response to the stressor—what the medical illness means to the patient, the pain and worry it may induce, and the dysfunction both mentally and physically, which may accrue from the physical malady present. The dysfunction may occur at work, in school, in relationships or in all three.

Consequently, it is a most important diagnosis for the medical setting and combines two genres of illnesses: the physical and the mental. There is little surprise that it is such a common diagnosis in the consultation–liaison patient group where the focus is on the psychological care of the medically ill [1]. Additionally, by having no metrics for the three essential diagnostic components it allows for AD to be employed quite liberally in the medical setting; and, it sets in motion the possibility that both psychotherapeutic and psychopharmacological interventions may help the patient deal with the circumstance they encounter.

What is less recognized is that AD may be a “way station” on the pathway toward a more serious mental disorder that can be interrupted or aborted if the psychological dysfunction and distress can be identified early. One might say the AD diagnosis is a most important ally and asset in the consultation–liaison setting despite its problems with validation and reliability, as it can be an early indicator of a developing major affective disorder or an anxiety disorder which has not yet met full criteria.

## 3. Psychobiology of AD

Understanding the psychobiology of AD may offer biological markers for a more reliable and valid diagnosis, and move AD from being characterized as sub-threshold entities. Since ADs are a result of stress—an essential part of the diagnosis—understanding the effects of stress would be an important starting point for understanding the psychobiology of AD. McEwen has carefully described the biological changes and consequences of stress over the past several years (McEwen 2015B) [13]. From his carefully studied research efforts it is known that there are significant consequences from stress on the central nervous system (CNS). As McEwen and Rasgon elegantly describe in an important discussion of the Brain and Body on Stress-Allostatic Load and Mechanism of Depression and Dementia, dysregulation of neuronal architecture and function take place as well as physiological dysregulation (McEwen and Rasgon 2018) [13]. As these authors point out, stress is a major factor in psychiatric illness, and the brain is the key organ of the stress response because it determines what is threatening and therefore stressful, and also controls the behavior and physiological responses.

McEwen and Rasgon describe that the concept of allostatic load embodies the idea that the dysregulation of brain-body interactions by life experiences and health-related behaviors can result in systemic pathophysiology and changes in the CNS that are associated with psychiatric disorders (McEwen and Rasgon 2018) [14]. Allostatic load refers to the wear and tear on the body that results from either too much stress or from inefficient management of the stress, for example, not stopping unnecessary functions. Allostasis literally means “*achieving stability through change”*. This can be mediated by adrenaline, noradrenaline, gluco-corticoids, pro- and anti-inflammatory cytokines, among other agents, and these entities can affect each other as well.

Stress can be biphasic—protection versus damage. McEwen and Rasgon explain that a good example of the biphasic actions of stress, e.g., protection versus damage, is in the immune system in which an acute stressor activates an acquired immune response mediation by catecholamines, gluco-corticoids, and locally produced immune mediators; yet chronic exposure to the same stressor over several weeks has the opposite effect and results in immune suppression [14,15,16].

McEwen et al. have also shown that adaptive changes in the hippocampus via cellular and molecular mechanisms may result from acute and chronic stress [17]. The hippocampus atrophies with chronic stress, in major depression, type 2 diabetes, post-traumatic stress disorder, chronic inflammation, and from lack of exercise. It increases in size with antidepressant therapy, regular exercise, and intense learning (McEwen and Rasgon 2018) [14]. McEwen has also reported that peptide/protein hormones, e.g., insulin-like growth factor 1(GF-1), insulin, ghrelin and leptin, may affect the hippocampus and therefore can be significantly involved in the reactions to stress. Stress has important and other yet to be determined effects on the CNS, and may be operative in the biology of the AD phenomenon.

## 4. AD with Depression and its Relationship to Major Depressive Disorder (MDD)

Since AD is such an important diagnosis in the liaison setting, and since AD with depression is one of the most commonly employed diagnoses, it would be important to understand if AD with depression is similar in its etiology, course, evolution, and treatment to that of a MDD. Post has proposed that MDD is a recurrent and progressive illness in need of long-term prevention [18]. Does AD with depression come more frequently, is it more difficult to treat with increasing episodes, or does it occur without a stressor with later episodes? Finally, does it follow the pernicious course of MDD with inadequate treatment? The long-term course of AD with depressive mood has not been sufficiently studied, whereas it has been observed with MDD that with repeated reoccurrences and inadequate treatment the episodes come more frequent, there is enhanced treatment refractoriness, cognitive dysfunction, and later episodes are not necessarily accompanied by a precipitating event. Unfortunately, in the liaison-psychiatry setting there is often no opportunity to see the patient after they leave the hospital or even the physician’s office, so the potential long-term effect of AD has not been adequately studied.

## 5. The Psychobiology of AD

Certain characteristics and behavior of AD in the liaison setting need to be understood. Are ADs different in the presence of a medical abnormality than in a patient with only mental symptoms? Could the biology of AD assist in diagnosis and treatment in the liaison setting? Essential research regarding AD as mentioned above has been attenuated because of the difficulty of obtaining comparable patient samples for investigation—and, in particular those with medical illness and those without. Meanwhile the American Psychiatric Association in DSM-5 describes a new chapter of diagnoses: *Trauma and Stressor Related Disorders* (DSM-5 2013). Strain and Friedman have reviewed the psychobiology of AD in a previous report [19] As said in this earlier report, an important approach to *Trauma and Stressor Related Disorders* origin in the liaison setting may be approached through examining the work of Hans Selye, who described the key role of the HPA system in the human stress response.

Therefore, an important question for the liaison psychiatrist would be to understand the HPA functions in MDD and in the *Trauma and Stressor Related Disorders*: post-traumatic stress disorder (PTSD), acute stress disorder (ASD), AD, and prolonged grief. Consequently, it would be important to know how the HPA system operates in AD and whether each AD subtype displays similar psychobiology alterations, or whether their psychobiology is more similar to the parent mood state, e.g., depression, anxiety. Since HPA changes are commonly observed in chronic stress syndromes, the overarching constructs of allostatic load and cumulative physiologic effects of repeated, even minor stressors, are a useful heuristic for understanding the psychobiology of AD and its effects in the medical setting. If AD with depression was similar psychobiologically to MDD, then treatments for the latter may apply to the former. A similar issue could prevail between AD with anxiety and anxiety disorders, and thereby effect suggestions for treatment for the former. This would be extremely helpful for the liaison psychiatrist in their approach to treatment. In any event, the relationship between trauma and stressor related disorders and chronic stress is an important avenue to understand these least researched psychiatric entities including AD.

## 6. The Psychobiology of Resistance

Finally, an understanding of the biology of resilience may highlight important understandings of the changes that may occur in the recovery from AD. Improvement in mental performance after treatment of AD may well be reflected in many of the same biological changes that occur with resilience. Resilience is management and recovery from psychopathology, e.g., reduction in the stressor, reduction in dysfunction, and reduction in distress or dysphoria, all critical for the diagnosis of AD and its treatment in the liaison setting. Do the same genetic differences determine vulnerability versus resilience in MDD and trauma and stressor related disorders including AD? This would be important for the liaison psychiatrist to know.

Southwick and Charney have elucidated the psychological tools for enhancing resilience and the biological correlates which are affected with acute stress (Southwick and Charney [20]. This insight may aid our understanding of the improvement of AD with treatment, and overtime in liaison psychiatry. Certainly in the medical setting, with an improvement in the physical disease, the stressor—the physical disease—may no longer have a pernicious effect and act as a stressor. The patient returns to a state of normalcy with an elimination or diminution of the causative agent—the stressor—the physical illness. Moreover, it is well known that many individuals with even extreme stressors never develop PTSD, ASD, AD, or prolonged grief responses. Since AD may be incited by traumatic or non-traumatic stress, and the biological changes are consequences of the stressors, then psychotherapy and psychopharmacology may have the result of restoring psychological well-being and the restoration of biological processes caused by stress, e.g., cortisol, dopamine, serotonin, etc. Southwick and Charney highlight the important biological dimensions that are altered by stress and the changes that occur with resilience. This would be important information for the liaison psychiatrist dealing with AD.

## 7. Conclusions

ADs are a most useful diagnosis in the liaison psychiatry setting. Their dependence on a stressor and a reaction to the stressor considered to be in excess of what would be expected in the culture and social setting of the patient, leaves the diagnosis arbitrary and subjective. Hopefully the HPA heuristic may provide an approach to understanding the psychobiological context which may guide research and clinical trials for the treatment of trauma and stressor related disorders including AD. Furthermore, such research may highlight the most effective treatment for AD, both psychologically and pharmacologically. Liaison psychiatry needs a diagnostic entity like AD to accommodate the reactions to the stressors that the patient encounters, one of which is medical illness and its consequences. It is an important vehicle to identify persons in need of mental health care who do not reach the symptom level of a primary psychiatric disorder. It is especially helpful to the medically ill, the military, and children, where the stressors they endure may result in distress and/or dysfunction. In addition, since it is an accepted official diagnosis in both our primary diagnostic lexicons, it has a diagnostic code which allows reimbursement for care and thus promotes access to caretakers by having medical insurance assistance. Liaison psychiatry’s intimate contact with medicine allows the passage of key psychological knowledge to migrate from one specialty to another. Hopefully more patients will be identified who could benefit from psychological management as they struggle with the stressors that have caused them anguish, dysphoria, and distress.
